# Insights into nanoparticle surface bonding and coating architecture via multinuclear NMR

**DOI:** 10.20935/acadnano7737

**Published:** 2025-05-29

**Authors:** Jacob D. Aubrey, James Gibson, John T. Leman, Benjamin M. Yeh, Peter J. Bonitatibus

**Affiliations:** 1 Department of Chemistry and Chemical Biology, Renssalaer Polytechnic Institute, Troy, NY 12180-3522, USA.; 2 Department of Radiology and Biomedical Imaging, University of California San Francisco, San Francisco, CA 94143-0628, USA.

**Keywords:** tantalum oxide, nanoparticle, siloxane coating, diagnostic imaging agent, X-ray computed tomography, ^29^Si NMR

## Abstract

Tantalum oxide nanoparticles (TaO_x_ NPs) are promising as high-*Z*-contrast agents for computed tomography (CT) due to their profound imaging benefits relative to those of clinical iodinated contrast media (ICM) at the X-ray tube voltages ≥100 kVp required for most patients. Furthermore, TaO_x_ NPs have prevailed through extensive non-GLP and GLP (good laboratory practice) preclinical development, including in vivo/vitro safety testing and imaging efficacy studies. This is due in part to innovative structural engineering of the NPs’ core size and coating, which has been shown to provide favorable pharmacokinetics and promote rapid renal clearance, with negligible organ retention. In this study, a carboxybetaine zwitterionic siloxane polymer (CZ) coating for a lead candidate TaO_x_ NP is thoroughly characterized using multinuclear/multidimensional nuclear magnetic resonance (NMR) spectroscopic techniques. ^1^H and ^1^H/^13^C heteronuclear multiple bond correlation NMR spectroscopies are used to confirm the CZ coating’s structure, and in combination with ^29^Si NMR, the architecture of the siloxane coating bound to the TaO_x_ NPs’ surface is described. Of particular significance, ^29^Si NMR spectra were used to identify the T-region bonding modes of the CZ coating and show the superiority of diafiltration over dialysis for purification of the TaO_x_ NPs. Through a spectral comparison, a cyclic siloxane impurity in the TaO_x_ NP product purified through dialysis was found to be absent in the product purified through diafiltration. Finally, the ^1^H Carr–Purcell–Meiboom–Gill (CPMG) NMR pulse sequence was used in a novel manner to probe the distance-dependent interactions between the ^1^H spins of the CZ coating and the TaO_x_ NPs’ surface.

## 1. Introduction

Clinical iodinated contrast media (ICM) for computed tomography (CT) imaging are termed “extracellular extravascular” agents [1-5] and all share several limitations. The rapid nonspecific redistribution or equilibration of small-molecule ICM from plasma to interstitial fluid following their injection results in the rapid loss of the intravascular CT signal needed to visualize complex anatomies and pathologies [6-15]. This reduction in contrast presents a compounded problem when imaging in populations of large/obese patients due to the mismatch between iodine’s K-edge (33.2 keV) and the high kVp needed to image these comorbidity-prone populations [6-8, 10, 16-22]. Indeed, the prevalent X-ray tube voltage used in clinical settings (120 kVp) results in an average X-ray energy of 81 keV [11], a value well above iodine’s K-edge. Moreover, ICM show a similar appearance to vascular calcifications or metal implants such as stents [23-27]. Importantly, all ICM show cross-reactivity such that patients intolerant to one iodinated agent are at increased risk of reactions to the entire class [12, 28-35]. Despite these shortcomings, ICM remain the only FDA-approved intravenous contrast agents for CT imaging; no substantively new injectable CT contrast agent has been introduced in over 50 years [36-38].

For over a decade, heavy research into the use of tantalum oxide nanoparticles as a new CT contrast agent has been performed to address the limitations of ICM [11, 12, 39-55]. After extensive non-GLP and GLP (good laboratory practice) preclinical development, safety tests, and imaging efficacy studies, carboxybetaine zwitterionic siloxane polymer (CZ)-coated tantalum oxide nanoparticles (TaOx NPs) have emerged as a lead candidate design [8, 10, 36, 44, 45, 56-58]. As the high-*Z* reporter element, Ta (Z = 73) has a higher-energy K-edge (67.4 keV) than that of iodine (33.2 keV) that attenuates the X-ray spectrum generated by the tube potentials (kVp) used in clinical settings better and can effectively address the challenges of the longer path lengths and beam hardening that present in patient populations with obesity. Compared to ICM, we have demonstrated that CZ TaO_x_ NPs are superior to iodine in terms of their conspicuity in vivo in the hepatic vasculature in swine [10] and the thoracic arteries in rats [8] and rabbits [45]. Innovative structural engineering and chemical manipulation of tantalum oxide’s core size (~3 nm) and the CZ coating have been shown to control the agent’s (bulk solution) physicochemical properties, such as its viscosity and osmolality, and pharmacokinetics and to promote rapid renal clearance, with negligible organ retention [6-8, 36, 39, 56, 57]. The CZ coating on the TaO_x_ NPs imparts water solubility; is stable within an exceptionally wide range of pH and autoclaving conditions [8]; and features distributed charges yet is charge-neutral overall, thereby avoiding the potential cytotoxicity concerns associated with cationic coatings [36, 40, 57, 58]. To advance the clinical translation of CZ TaO_x_ NPs further as an intravenous contrast agent for improved diagnostic CT imaging capabilities, understanding the surface bonding and coating architecture afforded by their CZ coating, as well as the impact of the agent purification processes, through rigorous characterization is required.

Nuclear magnetic resonance (NMR) spectroscopy is a robust technique with a wealth of literature surrounding its use in the NP arena, including 1D ^1^H NMR to confirm ligand attachment and quantitative 1D ^1^H NMR to determine the metal-to-ligand ratio in NPs [59]. Two-dimensional NMR techniques such as heteronuclear single and multiple quantum coherence (HSQC and HMQC), or heteronuclear multiple bond correlation (HMBC), can be used to confirm the overall particle structure, as well as the purity of the NP products [8, 59-64]. Diffusion-ordered spectroscopy (DOSY) has been utilized to calculate the ligand diffusion coefficients and the hydrodynamic diameters of NPs [59, 65, 66], while nuclear Overhauser effect spectroscopy (NOESY) has many applications in the characterization of NP coatings, including categorization of the ligand coating patterns as Janus, patchy/striped, or randomly mixed [67, 68]. In this article, we report the use of 1D ^1^H NMR to confirm that the CZ coating is bound to the slow-tumbling nanoparticles and the use of 2D ^1^H/^13^C HMBC NMR to confirm the ligand structure and the improved purity of the NPs relative to these properties in prior work [8]. Solution-state ^29^Si NMR provides insights into and a deeper understanding of the siloxane polymer architecture that constitutes the CZ TaO_x_ NPs through the distribution of the resonances observed in the T-region. The ^29^Si spectra also show that diafiltration is the preferred NP purification process compared to dialysis, with the removal of a small impurity tentatively assigned as a cyclic siloxane. Lastly, the Carr–Purcell–Meiboom–Gill (CPMG) NMR pulse sequence [69, 70] was investigated to evaluate the distance-dependent interactions between the ^1^H spins of CZ and the TaO_x_ NPs’ surface.

## 2. Materials and methods

### 2.1. Synthesis and purification

The CZ TaO_x_ NP batches were prepared using a previously described methodology [8] with the following modifications: the tantalum ethoxide (CAS No.: 6074-84-6), Ta(OEt)_5_, was obtained from City Chemical (SKU TC1002-BULK) as 2 kg lots and distilled prior to its use; the input dimethylaminopropyltrimethoxysilane (CAS No.: 2530-86-1, Gelest SID3547.0, Gelest Inc., Morrisville, PA, USA) was used as received (and not distilled); the synthesis solvent was replaced with 2-butanol (CAS No.: 78-92-2, anhydrous, 99.5%, Sigma-Aldrich 294810, Sigma-Aldrich Inc., St. Louis, MO, USA); and the scale was increased to 1.1 kg per batch (Ta(OEt)_5_ basis) in a 50 L cylindrical reactor (Chemglass, CG-1972, Chemglass, Vineland, NJ, USA). Tantalum ethoxide was distilled prior to its use (150 °C, 375 mTorr, using Vigreux and condenser columns measuring 1 m, at a 10.25 kg scale). Distillation has previously been performed at a higher pressure, of ~1 Torr, but required a higher temperature of 215 °C. A short path from the Vigreux column to the receiving vessel was used, as the high-purity Ta(OEt)_5_ may have solidified (and plugged) en route to its collection otherwise. The full details of all synthetic modifications [8], including the reagent ratios, may be disclosed by the corresponding author on request while a forthcoming manuscript (discussing the impact of these details on favorable results from in vitro immunotoxicological studies) is in preparation [58]. Ultrapure water for synthesis and purification was prepared using a MilliporeSigma Milli-Q IQ 7000 (St. Louis, MO, USA) water purification system equipped with IPAK Meta and IPAK Quanta cartridges and a Biopak polisher for pyrogen-/nuclease-/protease-/bacteria-free ultrapure water. Purification of CZ TaO_x_ NP batches through dialysis previously utilized Snake Skin (Thermo Fisher Scientific, Waltham, MA, USA) regenerated cellulose tubing and a standard (static) dialysis setup [8]. In the current work, the Spectra/Por^®^ dry RC dialysis tubing, with a 3.5/6–8 kDa MWCO (54/50 mm, 50/100 ft), was part of the dynamic dialysis setup, which involved 5 L process dialysis tanks (in series), as provided by Repligen Corporation. The details of diafiltration, including the system provided by Pall, the cartridges, the MWCOs of the membrane stack, and the relevant processing volumes and endpoint parameters, may be disclosed by the corresponding author on request while the aforementioned manuscript is in preparation. Briefly, the diafiltration system was a Pall Centramate system configured with Masterflex PharmaPure (24) tubing; the first diafiltration step required processing against low-kDa or low-molecular-weight-cutoff (MWCO) polyether sulfone (PES) stacked membranes (Pall Omega Centramate T-series 0.1 m^2^) to remove low-MW particles, e.g., chloride and bromide ammonium salts, etc. The second step was a straightforward ultrapurification step that used a larger-MWCO PES membrane stack (Pall Omega Centramate T-series 0.1 m^2^) to remove a vanishingly small population of particles >5 nm.

### 2.2. Sample analyses

Multiple batches of CZ TaO_x_ NPs were prepared (7) at the 50 L scale and characterized for batch-to-batch reproducibility. While thorough characterization was described previously [8], we include additional characterization data for these (7) large-scale batches. The hydrodynamic size of the particles was measured through DLS at 25 °C in water (without the addition of PBS) using a Brookhaven 90Plus nanoparticle size analyzer. Hydrodynamic sizes of 3.1 ± 0.2 nm (mean diameter) were measured based on the Z_effective_ values of the volume-weight-based distributions for the batches. A Malvern Zetasizer Nano ZS instrument was used to measure the zeta potential at 25 °C. Measurements were made at a pH of 5.6 and a pH of 7.4 after adjustment using 1 N standardized NaOH. The instrument was validated by running an appropriate standard (Zeta Potential Transfer Standard, DTS0050, with a zeta potential value of −42 ± 4 mV at 25 °C, Malvern Instruments) before all of the zeta potential measurements. The zeta potential for the CZ TaO_x_ NPs was −1.9 ± 0.9 mV at a pH of 5.63 and −9.0 ± 5.6 mV at a pH of 7.04. The viscosities of the CZ TaO_x_ NP formulations were measured at 37 °C using a portable viscometer, the microVISC^™^ (Rheosense, San Ramon, CA, USA), after validating the data collection for standard solutions of water and isopropyl alcohol. The osmolalities were measured using a Wescor Vapro (ELITech, Logan Utah, UT, USA) after measuring osmolality standards in triplicate. The viscosities of the CZ TaO_x_ NP solutions formulated to 244 mg of Ta/mL were measured as 9.56 ± 0.2 cP at 37 °C; meanwhile, the osmolalities were observed to be 748 ± 10 mOsm/kg. We were unable to visualize these amorphous particles ~3 nm through TEM.

We performed an elemental analysis in a manner identical to methods previously published by our group [39] using ICP-OES quantification (Spectro Arcos, Kleve, Germany), which enabled us to determine the percent Ta and the percent Si (by weight) in the products and the percent yields of the products based on the mass of Ta used as the starting material, as well as the absolute concentrations of the unformulated and formulated nanoparticle solutions. ICP-OES was used directly to quantitatively determine the mg/g of both Ta and Si per aqueous sample, and these data were then used to calculate the Si/Ta mole ratios (1.40–1.42). The Si/Ta parameter is important in terms of assessments of batch-to-batch reproducibility and the QC specifications for a future product. Our technique for detecting the endotoxin present in the CZ TaO_x_ NP agent relies on an EndoSafe^®^ nexgen-PTS^™^ (Charles River Laboratories, Wilmington, MA, USA). We assume a clinically relevant injectable human dose of 500 mgTa/kg, injected at 300 mgTa/mL; therefore, at 10x the maximum valid dilution, CZ TaO_x_ NP solutions of 5 mgTa/mL need to pass 0.05 EU/mL sensitivity (EndoSafe^®^ PTS2005F cartridge, Charles River, Shrewsbury, MA, USA). All of the samples for in vivo evaluation pass, i.e., <0.05 EU/mL.

**Figure 1 •:**
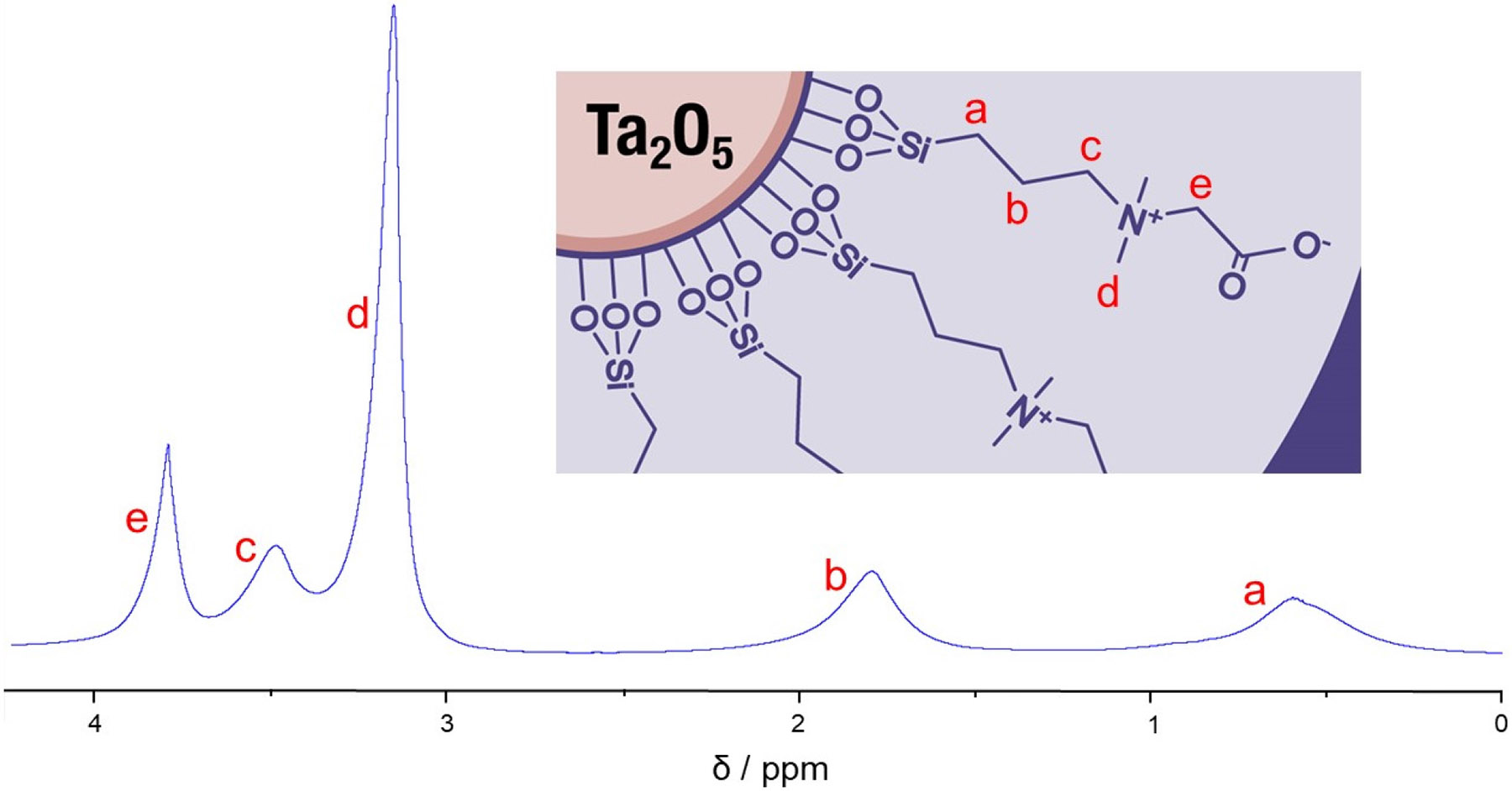
The ^1^H NMR spectrum of the 3 nm CZ TaO_x_ NPs in D_2_O. Letters *a–e* in the inset NP graphic denote ^1^H positions along the CZ backbone and correspond with peaks *a–e* in the NMR spectrum.

**Figure 2 •:**
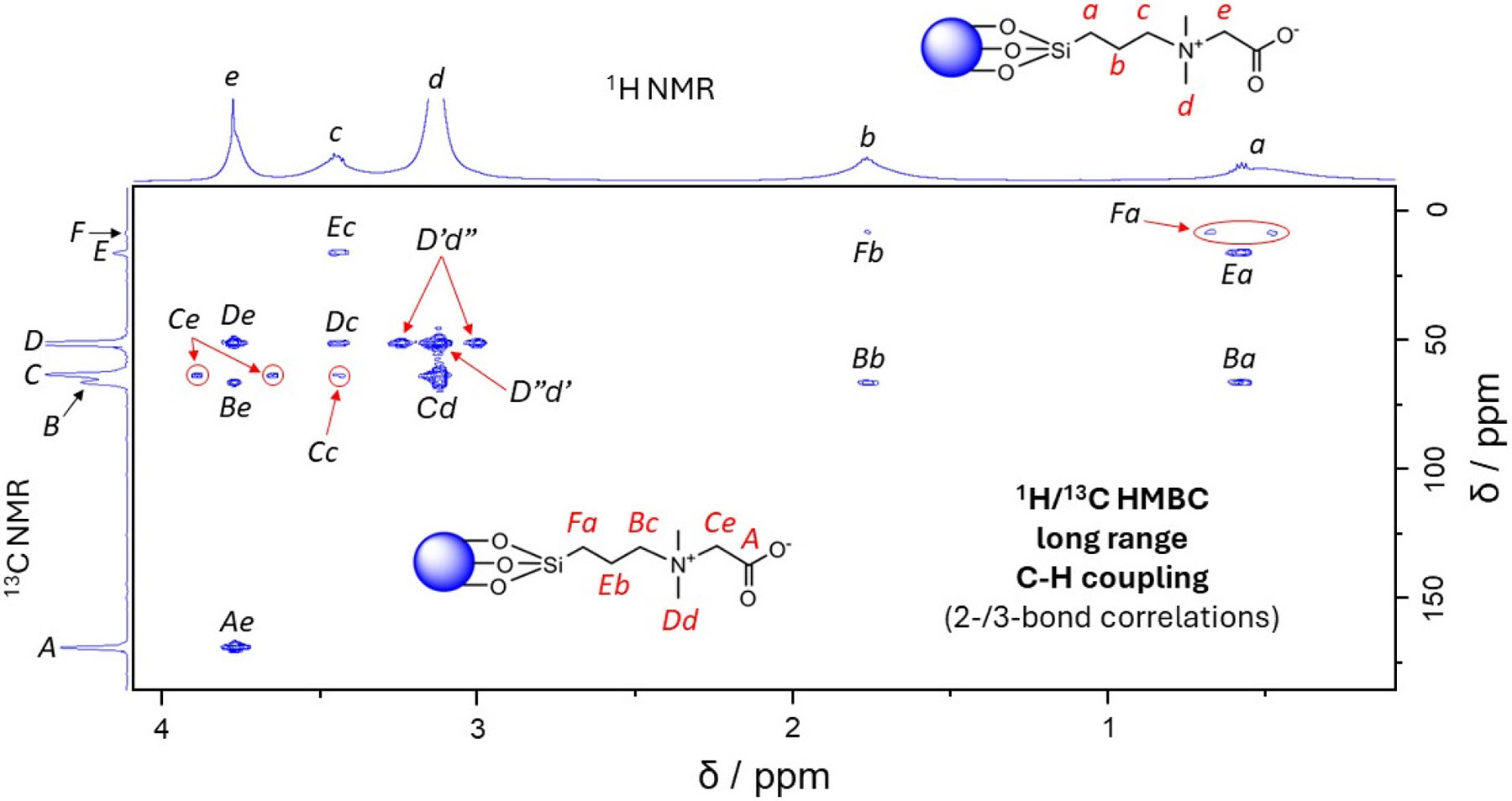
The HMBC spectrum for the CZ TaO_x_ NPs in D_2_O showing ^1^H (F1 axis) and ^13^C (F2 axis) 2-to-3 bond correlations. The red-letter labels *a–e* for the NP graphic at the top of the figure denote ^1^H positions along the CZ backbone and correspond with peaks *a–e* of the F1 axis. The red-letter labels *A–D* for the second NP graphic within the figure denote ^13^C positions along the CZ backbone and correspond to peaks *A–D*. Black letter pairings on the plot label peaks associated with ^1^H/^13^C interactions, e.g., label *Ae* in the bottom left-hand corner identifies the peak that arises from the correlation between ^1^H *e* and ^13^C *A*. Due to limitations of the low-pass *J*-filter, single-bond correlations, *Fa*, *Ce*, and *D’d”*, are observed [71].

All CZ TaO_x_ NP samples for NMR spectroscopy were prepared in D_2_O. The experiments were conducted on a Bruker 600 MHz (14.1 Tesla) wide-bore NMR spectrometer. All FID and spectral processing was conducted in Bruker’s TopSpin 4.4.0 software. The ^1^H NMR spectrum of the diafiltration-purified CZ TaO_x_ NPs was acquired using Bruker’s *zg* pulse sequence with a 1.5 s recycle delay, a 1.7 s acquisition time, a spectral width of 9.62 kHz, 32,768 data points, and 16 scans (Figure 1). An HMBC experiment was conducted on the diafiltration-purified CZ TaO_x_ NPs using Bruker’s *hmbcgplpndqf* pulse program (Figure 2) with a 1.5 s recycle delay and 4 scans. For the ^1^H nucleus, a 131 ms acquisition time, a 7.81 kHz spectral width, and 2048 data points were used. For the ^13^C nucleus, a 38.5 ms acquisition time, a 33.21 kHz spectral width, and 128 data points were used.

**Figure 3 •:**
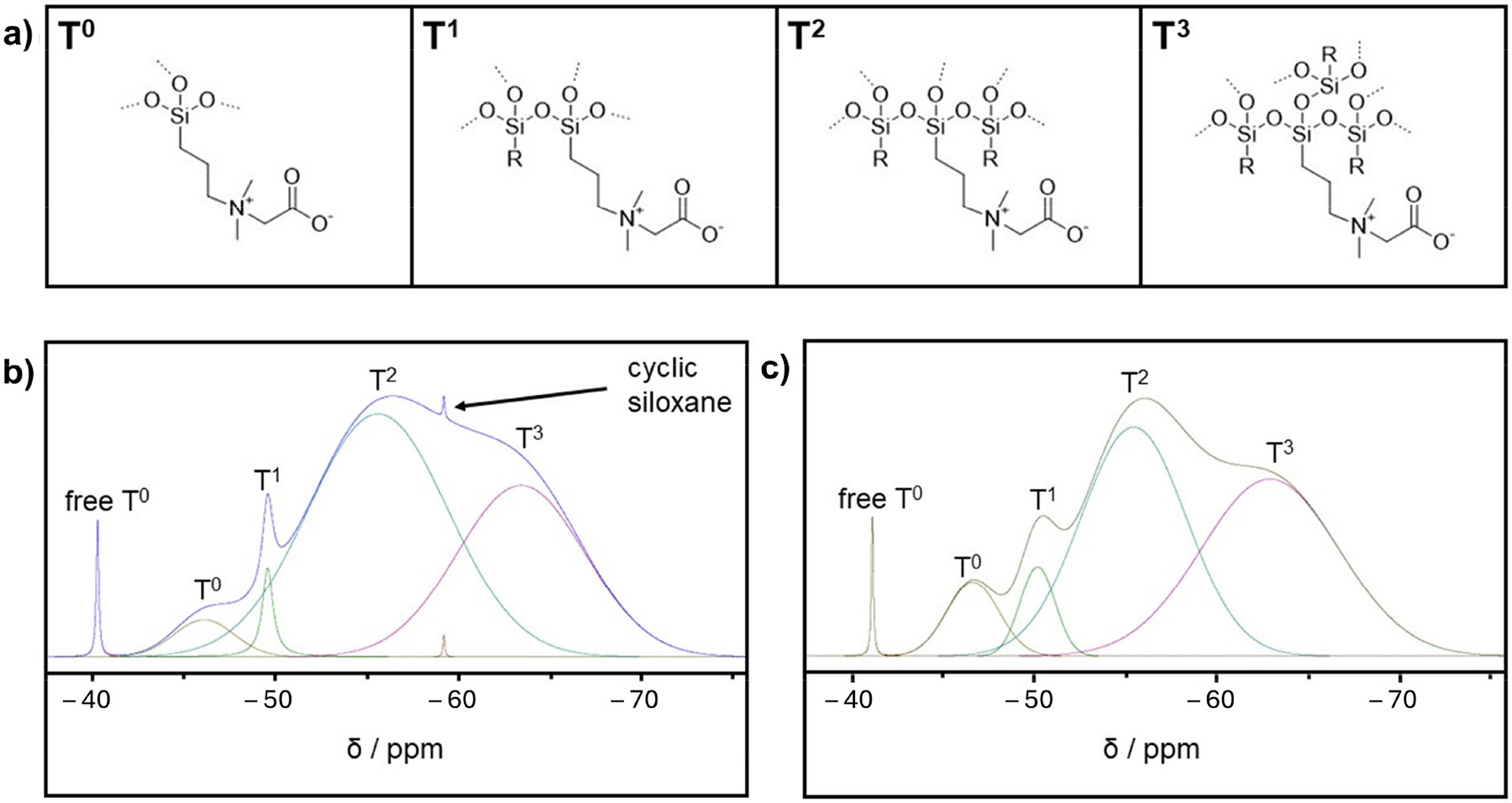
(**a**) Silicon T-structures with the T descriptor referring to the central Si atom featuring a fully drawn zwitterionic group. (**b**) Deconvoluted and curve-fitted ^29^Si NMR spectrum for dialysis-purified CZ TaO_x_ NPs. (**c**) Deconvoluted and curve-fitted ^29^Si NMR spectrum for CZ TaO_x_ NPs purified through diafiltration.

The ^29^Si NMR spectra were acquired for a dialysis-purified CZ TaO_x_ NP sample (Figure 3b) and a diafiltration-purified CZ TaO_x_ NP NMR sample (Figure 3c) using the same procedure. The recycle delay was 60 s, the acquisition time was 43 ms, the spectral width was 23.81 kHz, the number of data points was 2048, and the number of scans was 1024. ^1^H-decoupling was also employed. To quantify the different Si binding modes denoted by the various peaks, spectral deconvolution and curve fitting were performed in TopSpin for both spectra. The baseline tool was used to eliminate interference from the probe background prior to the deconvolution/fitting process for each sample. TopSpin’s deconvolution tool, which utilizes the estimated peak position, full-width-at-half-maximum (FWHM), and %gauss, was then used to determine the shapes and sum for each spectrum, as well as the peak area for each shape (Figure 3). These data were used to calculate the T percentages in Microsoft Excel^®^ for Microsoft 365 MSO (Version 2504 Build 16.0.18730.20186).

**Figure 4 •:**
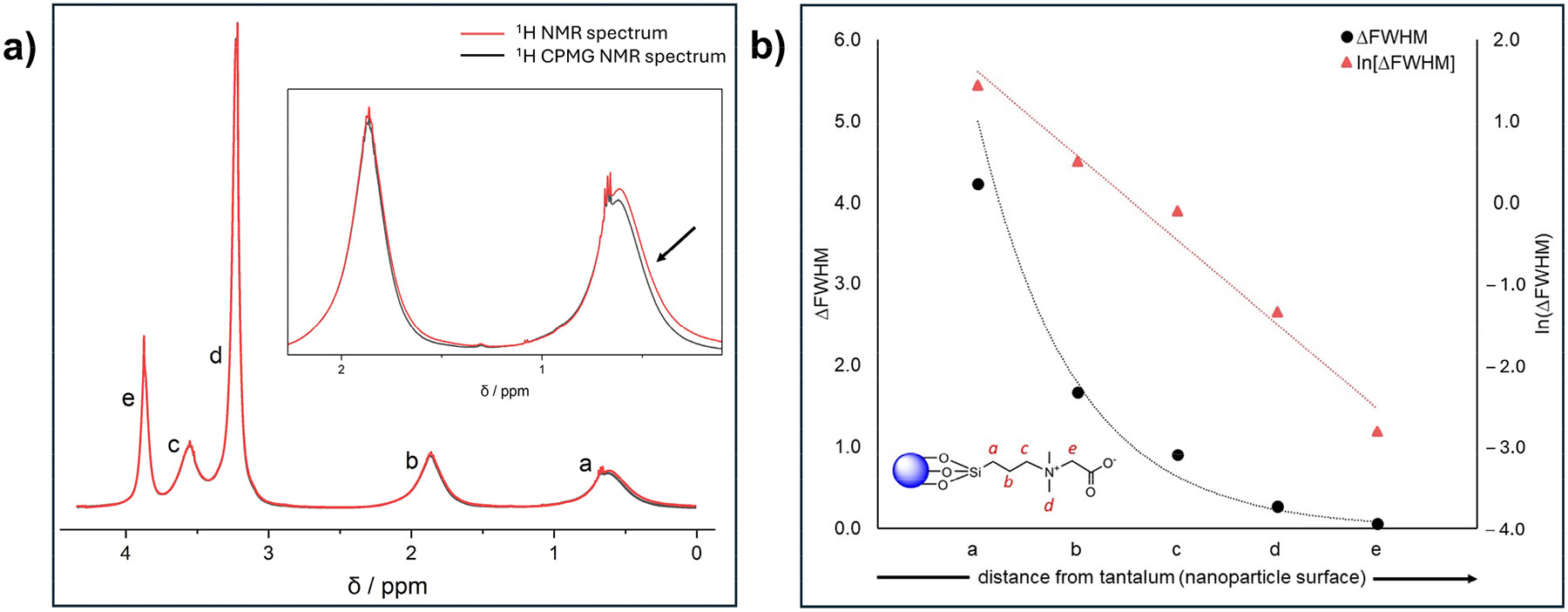
(**a**) Overlay of ^1^H NMR and ^1^H CPMG NMR spectra (in D_2_O); the inset highlights the CPMG-facilitated decrease in peak broadening (arrow). (**b**) Black circular data points: the ΔFWHM between the ^1^H NMR and ^1^H CPMG NMR spectra for resonances *a–e* (black points) vs. the peak assignment (line-fit, R^2^ = 0.9924). Orange triangular data points: natural log (*ln*) of the ΔFWHM for peaks *a–e* plotted vs. the peak identity (R^2^ = 0.9763).

The ^1^H NMR spectrum for the CZ TaO_x_ NPs was acquired using Bruker’s *zg* pulse sequence with a 10 s recycle delay, a 3.4 s acquisition time, a spectral width of 9.62 kHz, 65,536 data points, and 8 scans. Following this, the Carr–Purcell–Meiboom–Gill (CPMG) pulse sequence (τ = 20 μs; loop count = 20) was executed on the same CZ TaO_x_ NP sample to analyze the broadening decreases (narrowing) using the same spectral width, number of data points, and number of scans as those used for the ^1^H NMR experiment. After processing both FIDs in TopSpin, the refined spectra were exported into Origin as *x,y* coordinates and overlayed (Figure 4a) to visualize the peak narrowing. To quantify the narrowing, the FWHM was measured in TopSpin for each peak in each spectrum. Using Microsoft Excel, the change (Δ) in the FWHM for each peak (FWHM ^1^H NMR spectrum-FWHM CPMG spectrum) was plotted vs. the relative distance from the NP surface with an exponential decay line-fit applied to the data. Next, the natural log (*ln*) of the ΔFWHM was plotted onto the secondary *y*-axis vs. the same relative distance from the nanoparticle surface, and a linear fit was applied (Figure 4b).

## 3. Results and discussion

### 3.1. ^1^H NMR and ^1^H/^13^C HMBC

In the ^1^H NMR spectrum (Figure 1), there are five (5) peaks: *a* (δ = 0.62 ppm), *b* (δ = 1.86 ppm), *c* (δ = 3.56 ppm), *d* (δ = 3.23 ppm), and *e* (δ = 3.87 ppm). Each ^1^H resonance is heavily broadened, preventing *J*-coupling interactions and splitting patterns from being observed [72-76]. We attribute this broadening to two factors: (1) the NP is quadrupolar in nature (^181^Ta, *I* = 7/2), which induces electric field gradients facilitating rapid transverse relaxation [52], and (2) since the CZ coating is polymeric (exhibiting varying degrees of condensation) and anchored to the NP’s surface, motion varies from point-to-point or methylene-to-methylene and restricted overall resulting in uneven averaging of magnetic field inhomogeneities which also contributes to rapid transverse relaxation [49-51, 53]. Despite this broadening, peak assignments can still be made with high confidence by analyzing the chemical shifts while considering the structure of the CZ coating, as well as through the relative peak intensities. Previously, we reported the ^1^H, ^13^C, and ^1^H/^13^C NMR spectra for the CZ TaO_x_ NP product using underdeveloped synthesis and purification processes [8]. In those spectra, two (2) rather significant impurities were noted: (1) an isopropyl-ester-derivatized form of CZ TaO_x_ NP representing close to 10% of the product yield through peak integration and (2) a residual isopropyl alcohol (IPA) synthesis solvent which was responsible for the formation of the isopropyl-ester-derivatized CZ TaO_x_ NPs. In Figure 1, we see no evidence of the previous impurities, particularly the residual alcoholic synthesis solvent, due to developments in the synthetic modifications and chemical process (including scaling up by a factor of nearly 50x) that arrest the formation of the unwanted transesterified product. The ^1^H NMR spectrum is devoid of protic impurities and confirms both the coating’s structure and surface attachment. The peak assignments can be confirmed further by heteronuclear ^1^H/^13^C correlations in the HMBC 2D NMR spectrum (Figure 2). For example, the peak “*Ae*” establishes a connection between ^13^C carbonyl carbon “*A*” to the adjacent methylene protons “*e*” through the peak “*Ae*” [77]. It must be highlighted that although broadening washed out the *J*-coupling, the 1D and 2D data provide solid evidence that the CZ coating is indeed bound to the nanoparticle surface. In the HMBC spectrum, also note that the “self-cross-peaks” *Fa*, *Ce*, and *D’d”* arise from single-bond ^1^H/^13^C interactions that have slipped past the HMBC pulse sequence *J*-filter [71]. These features are identified in Figure 2, as are all features, cleanly resolved and securely identified.

### 3.2. ^29^Si NMR

In addition to the synthetic modifications identified above in section 2.1, the process of CZ TaO_x_ NP purification was revised. Diafiltration was investigated and ultimately implemented [36, 56-58] because static and dynamic dialysis processes are not scalable for production (at any reasonable scale), and we discovered it to be a superior, more thorough process of purification based on ^29^Si NMR data. ^29^Si has a non-zero magnetic moment, and its wide chemical shift dispersion makes it highly diagnostic for characterization purposes [78-82], a property we exploited to gain a deeper understanding of the CZ TaO_x_ NPs, specifically the siloxane polymer architecture enveloping the NPs. Through identification and quantification of different silicon environments, ^29^Si NMR revealed the highly crosslinked and complex nature of the CZ coating.

Figure 3a serves as a key to describe the “T-region” bonding modes observed for the CZ coating; note that T^0^ corresponds to an uncondensed silicon species, whereas T^1^–T^3^ describe various degrees of condensation or numbers of neighboring O3SiR groups bonded to a silicon atom. ^29^Si NMR spectroscopy was performed on the CZ TaO_x_ NP samples purified through dialysis (Figure 3b) and diafiltration (Figure 3c). Both spectra showed very similar profiles overall, with multiple overlapping and broad resonances in the T^1^–T^3^ region (−50 to −70 ppm), indicating the degree of condensation and confirming the presence of the NP-surface-bonded siloxane polymer [83]. The T^1^–T^3^ percentages for dialysis and diafiltration from the deconvolution/curve-fitting protocols were estimated to be 2% for T^1^ (−49.55 ppm), 57% for T^2^ (−56.93 ppm), and 36% for T^3^ (−62.65 ppm) and 6% for T^1^ (−50.03 ppm), 43% for T^2^ (−55.19 ppm), and 44% for T^3^ (−62.53 ppm), respectively, which accounted for ~94% of the total silicon in both cases. Insight into the reproducibility of these data is provided by the standard deviations in the T^1^–T^3^ percentages for the lead candidate CZ TaO_x_ NP contrast agents purified through diafiltration, 6% for T^1^ (SD = 0.56), 43% for T^2^ (SD = 1.11), and 44% for T^3^ (SD = 1.31), for a sample size N = 3. The amount of free uncondensed T^0^ for both purification processes was estimated to be 1%, resonating sharply and well resolved at −40.29 ppm and −41.14 ppm; the assignment as uncondensed silicon species hydrogen-bonded to the CZ coating agrees with previous reports [84]. The remaining ~5% of the silicon signal is tentatively assigned as uncondensed T^0^ zwitterionic silane, entrained or possibly bonded to the NPs’ surface given the unique resonance/shift observed at −45.61 and −46.54 ppm for the dialyzed and diafiltered products, respectively. We cannot rule out the bonding to the NPs’ surface as Si(OH)_3_(OTa)_3–x_ species.

By way of^29^Si spectral comparisons, an additional peak at −59.28 ppm was identified in the dialysis-purified sample (Figure 3b), corresponding to ~0.1% of the total silicon in the spectrum, that was absent in the ^29^Si NMR results for the CZ TaO_x_ NP product purified through diafiltration. We believe this surprising finding to be a cyclic siloxane impurity, *cyclo*-(SiO)_3_SiR or CZ-functionalized polyhedral oligomeric silsesquioxane (CZ-POSS, [RSiO_1.5_]_8_), based on its characteristic shift (−59.28 ppm) compared to data from the literature (−60.6 and −60.5 ppm) [83, 85]. The presence of this impurity in the dialysis-purified ^29^Si NMR spectrum (Figure 3b) is emphasized here due to its absence in the diafiltration-purified ^29^Si NMR spectrum (Figure 3c); this feature clearly shows the superiority of diafiltration over dialysis. There are many reports in the literature of cyclic siloxanes in addition to those identified above, including a series of cyclic siloxanes with ^29^Si NMR chemical shifts at −58.97, −59.69, −59.12, −59.00, and −58.46 ppm [86], to further substantiate the tentative assignment of this impurity in the dialysis-purified CZ TaO_x_ NPs, featuring a loose ionic association that is removed through diafiltration.

### 3.3. CPMG-facilitated peak narrowing

Here, we leveraged a ^1^H Carr–Purcell–Meiboom–Gill (CPMG) spin-echo pulse sequence to probe the distance-dependent interactions between the ^1^H nuclei of the CZ coating and the NPs’ surface. In effect, the CPMG sequence narrowed the resonances (recovered signals) of the ^1^H nuclei distributed along the zwitterionic chain of the CZ coating. To quantify the narrowing, the change in the FWHM (ΔFWHM) was calculated for each peak in the 1D ^1^H spectrum that resulted from the CPMG experiment. All methylene groups assigned *a*, *b*, *c*, and *e*, including methyl groups *d* (see Figure 1), featured increased peak narrowing and signal recovery due to the CPMG sequence, albeit this effect was dependent on the proximity to the NPs’ surface and ^181^Ta nuclei (Figure 4). The peak narrowing or ΔFWHM was most pronounced for peak *a*, which was closest to the NPs’ surface and ^181^Ta, but the signal recovery decreased as the ^1^H nuclei were distributed radially from the NPs’ surface (Figure 4b). The distance dependency was established through the exponential line-fit applied to the data (R^2^ =0.9924). The recovery of NMR signals through the application of spin-echo pulse sequences, specifically the CPMG sequence, has been demonstrated in paramagnetic systems [87]. While CZ TaO_x_ NPs contain quadrupolar nuclei (^181^Ta, *I* = 7/2) generally assigned a +5 oxidation state, the presence of unpaired surface spins may account for the experimental behavior observed. This observation may also be rationalized considering that (i) the strength of dipole–dipole interactions is distance-dependent [88] such that the magnitude of inhomogeneous line broadening is also distance-dependent and (ii) the ^1^H nuclei of the CZ coating are dipole–dipole-coupled with the ^181^Ta nuclei; therefore, the inhomogeneous line broadening due to ^181^Ta-^1^H dipolar coupling increases with proximity to the NPs’ surface (^181^Ta). The CPMG sequence was able to reverse the broadening and recover the signal loss in a distance-dependent manner; the trend observed through NMR indirectly corroborates previous data confirming that the CZ coating is bound to the NPs’ surface [8, 11, 39-41, 57].

## 4. Conclusions

In this article, we characterize the coating of our preclinical diagnostic CT imaging agent, CZ TaO_x_, in detail using multiple NMR techniques. Characterization of the CZ polymer using ^29^Si NMR provides a practical approach to evaluating the surface bonding across a wide range of nanoparticle systems with siloxane coatings. Solution ^1^H and ^1^H^/13^C HMBC NMR were used to reaffirm the structure of the CZ siloxane polymer, as well as to confirm the purity of CZ TaO_x_ NP batches from recent scale-up efforts. The updated synthesis and purification protocols eliminated the previously reported transesterified byproduct [8]. ^29^Si NMR was used to evaluate the CZ network and the bonding architecture though T-region percentage calculations; it was determined that T^2^ and T^3^ were the dominant bonding arrangements. The ^29^Si NMR spectral comparison between dialysis- and diafiltration-purified CZ TaO_x_ demonstrated the inability of dialysis to remove a cyclic siloxane impurity which was absent in the samples purified through diafiltration. Finally, by probing the CZ TaO_x_ NPs using the CPMG sequence, a clear, exponential relationship was noted between the FWHM of the ^1^H NMR CZ resonances and distance from the NPs’ surface.

## Data Availability

Data supporting these findings are available within the article, at https://doi.org/10.20935/AcadNano7737, or upon request.
